# Right‐Sided Dual‐Chamber Left Bundle Branch Area Pacing in Persistent Left Superior Vena Cava: A Case Report on Personalized Conduction System Pacing

**DOI:** 10.1155/cric/9833282

**Published:** 2026-08-17

**Authors:** Dhan Bahadur Shrestha, Ashlesha Chaudhary, Aamir Gilani, Jurgen Shtembari, James A. Storey

**Affiliations:** ^1^ Division of Cardiology, Department of Internal Medicine, Bassett Medical Center, Cooperstown, New York, USA, bassett.org; ^2^ Department of Internal Medicine, Bassett Medical Center, Cooperstown, New York, USA, bassett.org; ^3^ Division of Cardiology, Department of Internal Medicine, Carle Foundation Hospital, Urbana, Illinois, USA, carle.org; ^4^ Division of Electrophysiology, Department of Internal Medicine, Memorial Medical Center, Las Cruces, NM, USA, memorialmedical.com

**Keywords:** atrioventricular block, cardiac implantable electronic device, conduction system pacing, congenital venous anomaly, physiologic pacing, venography

## Abstract

**Background:**

Persistent left superior vena cava (PLSVC) can complicate transvenous device implantation due to challenging venous anatomy and limited sheath support during physiologic pacing lead delivery.

**Case Summary:**

A woman in her mid‐40s evaluated for a syncopal episode had ambulatory electrocardiography showing high‐grade atrioventricular block with prolonged pauses, deemed irreversible, leading to plans for a permanent pacemaker. Intraprocedural venography identified a PLSVC. A right‐sided dual chamber left bundle branch area pacing (LBBAP) was successfully performed. The procedure was uncomplicated, and follow‐up with cardiology was arranged.

**Discussion:**

Right‐sided LBBAP can be feasible and associated with positive outcomes in patients with PLSVC, and it should be considered a strong option when traditional venous routes are difficult. Early detection and adaptable pacing strategies are essential for maximizing outcomes in such patients.

**Take-Home Messages**

• Right‐sided LBBAP can provide a practical, physiological pacing option in select patients with PLSVC when traditional left‐sided lead placement is challenging.
• Right‐sided LBBAP is a feasible and effective strategy in patients with PLSVC, in the absence of structural heart disease, and can be achieved using standard sheaths in selected cases.
• Physiologic pacing should be prioritized in younger patients with high‐grade atrioventricular block to preserve ventricular synchrony and avoid long‐term pacing‐induced cardiomyopathy.

## 1. Introduction

Persistent left superior vena cava (PLSVC) is a rare congenital thoracic venous anomaly in which the left‐sided superior vena cava persists and typically drains into the coronary sinus and then the right atrium [1, 2]. A recent systematic review and meta‐analysis of 18 studies involving 169,835 individuals reported a pooled prevalence of 0.61% (95% confidence interval, 0.38%–0.89%) in populations not selected for congenital heart disease. Bilateral superior vena cava was identified in 0.32% of the population, isolated PLSVC in 0.10%, and a bridging brachiocephalic vein in approximately 0.11% [3]. Although usually asymptomatic and hemodynamically benign, PLSVC can create major technical challenges during transvenous device implantation because of its abnormal venous course, acute angulations, and limited sheath support for lead delivery [4, 5, 6]. These challenges are especially relevant for left bundle branch area pacing (LBBAP), which requires stable, perpendicular alignment of the delivery sheath against the interventricular septum for adequate septal penetration and capture of the conduction system, a system designed for a left‐sided approach. In patients with PLSVC, conventional left‐sided access through the coronary sinus may be difficult and compromise lead positioning and stability. In contrast, right‐sided venous access may provide a more favorable trajectory for physiologic pacing.

Here, we report a case of right‐sided dual‐chamber pacemaker implantation with LBBAP in a woman with high‐grade atrioventricular block and PLSVC with bridging of the left subclavian to the right brachiocephalic vein using a standard lumenless lead designed for left‐sided LBBAP. She subsequently needed personalized programming of her device to meet her physiological needs, given that postpacemaker implantation she continued to have symptoms suggestive of pacemaker syndrome.

## 2. Case Report

### 2.1. History of Presentation

A woman in her mid‐40s visited the cardiology clinic with 4 weeks of exertional chest discomfort, fatigue, intermittent palpitations, and a syncopal episode. She described the chest discomfort as a pressure‐like sensation localized to the left side, nonradiating, and exacerbated by physical activity. The palpitations were intermittent and described as a fluttering sensation in the chest. She denied any known relieving factors. Although engaging in moderate exertion at work, she experienced sudden fatigue followed by a transient loss of consciousness without any preceding symptoms such as chest pain, dizziness, or palpitations. The syncopal event was brief, and she regained full consciousness spontaneously. Witnesses noted no abnormal movements, rigidity, or postictal confusion. She denied associated symptoms such as dyspnea, diaphoresis, or nausea. Prior to symptom onset, she had no limitations to activity and maintained an active lifestyle.

On presentation, her heart rate was 56 beats/min; other vital signs were normal. She appeared euvolemic. Cardiovascular exam showed a regular rhythm without murmurs, rubs, or gallops. Pulmonary, abdominal, and neurologic exams were unremarkable.

### 2.2. Past Medical History

Her medical history includes patent ductus arteriosus closure at age 8 with no follow‐up until now. She was diagnosed and treated with Lyme disease in the past. No hypertension, diabetes, or other chronic illnesses were reported. Family history is unremarkable. She is married, employed, active, and socially drinks alcohol. She does not use tobacco or recreational drugs and has no known allergies.

### 2.3. Differential Diagnosis

The patient′s clinical picture, including exertional chest pressure, palpitations, fatigue, and exertion‐associated syncope, necessitated a broad differential diagnosis. The differential diagnosis included tachyarrhythmia, bradyarrhythmia from conduction system disease, myocardial ischemia, reflex syncope, orthostatic hypotension, conduction pathology, electrolyte imbalances, and medication‐induced AV block. Initially, arrhythmic causes such as tachyarrhythmias were considered, but telemetry failed to identify any episodes. Continuous monitoring showed significant bradyarrhythmias like 2:1 AV block and intermittent complete heart block, aligning with symptoms and indicating conduction system disease.

Cardiac ischemia was considered due to the exertional nature of her chest discomfort, but this was excluded through nonischemic ECGs, normal troponin, and cardiac MRI without late gadolinium enhancement. Reflex syncope and orthostatic hypotension were less likely due to the absence of prodrome and positional triggers. Reversible conduction disturbances from infectious or inflammatory etiologies were excluded through negative Lyme serology and a normal cardiac MRI. Electrolyte abnormalities and medication effects were ruled out through history and lab investigations. Consequently, a diagnosis of idiopathic nonreversible high‐grade AV block was established as the cause of her symptoms.

### 2.4. Investigations

**Figure 1:**
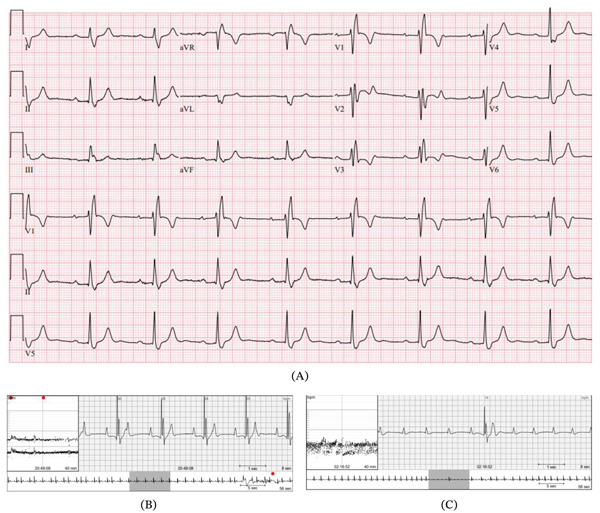
(A) Electrocardiogram at presentation showing sinus bradycardia with first‐degree atrioventricular block, and event monitor tracings demonstrating (B) intermittent 2:1 atrioventricular block and (C) complete heart block with pauses up to 4.5 s.

Electrocardiography revealed sinus bradycardia, first‐degree AV block (PR interval 240 ms), and right bundle–branch block (Figure 1A). A 2‐week monitoring period documented sinus bradycardia, Mobitz Type I, 2:1 conduction (Figure 1B), and intermittent complete AV block (Figure 1C), with pauses lasting up to 4.5 s and heart rates below 40 bpm for 13% of the recording duration.

Lyme serology was negative. Transthoracic echocardiography showed mild concentric left‐ventricular hypertrophy, preserved ejection fraction (EF), and septal motion abnormalities consistent with conduction delay, whereas right‐sided chambers and valves were normal and there was no pericardial effusion. Cardiac magnetic resonance imaging (MRI) confirmed structurally normal biventricular anatomy, no late gadolinium enhancement, and normal parametric mapping values. Routine laboratory testing, including a complete blood count, metabolic panel, thyroid‐stimulating hormone, and high‐sensitivity troponin I, was within normal limits.

### 2.5. Management

**Figure 2:**
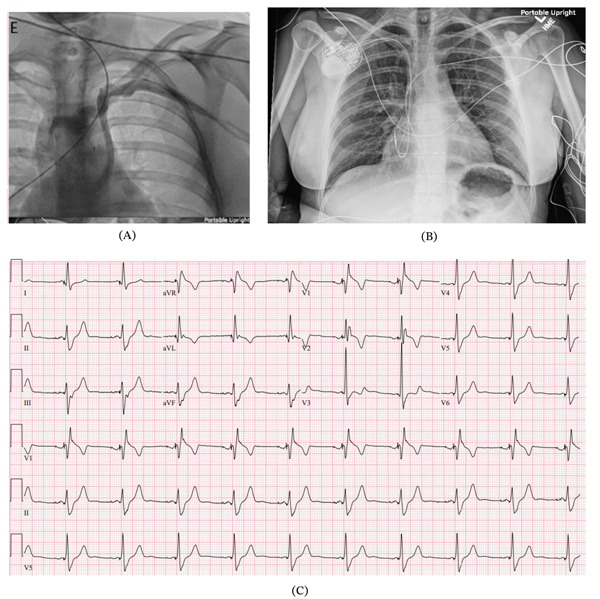
(A) Fluoroscopic image showing PLSVC with communication to the right SVC. (B) X‐ray showing appropriate lead positions following right‐sided approach for dual chamber pacemaker with LBBAP pacing ventricular lead. (C) A‐V sequential paced rhythm following dual chamber pacemaker with LBBAP pacing ventricular lead mimicking intrinsic rhythm.

Because of symptomatic high‐grade atrioventricular block, the patient received a dual‐chamber pacemaker under conscious sedation. Intraprocedural venography before pocket creation showed a PLSVC with communication at an acute angle to the right superior vena cava (Figure 2A). Given the acute angle of the connecting channel to the right brachiocephalic vein, we attempted right‐sided venography for a right‐sided approach to avoid the long course going retrograde from the coronary sinus and presumed difficulty in engaging the conduction system with such an approach. A right‐sided venography revealed normal venous anatomy.

A right prepectoral pocket was created using a standard technique. An atrial lead was advanced through axillary venous access and placed in the right atrial appendage. Using a second axillary venous access, a lumenless 3830 bipolar pacing lead was inserted into the mid interventricular septum for LBBAP (Figure 2B). Pacing was successful, with a QRS duration of 130 ms and an acceptable left ventricular activation time (Figure 2C). The lead was stable, and there were no immediate complications.

The device was initially programmed to DDD 60 bpm with an upper tracking rate of 145 bpm, a sensor‐driven upper rate of 135 bpm, and a postventricular atrial refractory period (PVARP) of 250 ms. The initial parameters were set relatively conservatively to ensure reliable pacing and decrease early postoperative arrhythmias. Early cardiac monitoring after the implant demonstrated continuous ventricular pacing as expected in her case of advanced AV block with stable lead parameters. The patient had an uneventful postoperative course and was discharged the following day.

### 2.6. Outcome and Follow‐Up

At 3 months, she reported palpitations and fatigue, particularly with physical activity. Pacemaker interrogation showed normal lead function, atrial pacing around 59%, 100% ventricular pacing, a brief episode of atrial tachycardia, and otherwise normal function. To help her heart rate increase appropriately with exercise, the device was changed from DDD mode to DDDR mode at a base rate of 60 bpm. After this change, she began experiencing increased chest discomfort and fatigue during exertion. At this point, we assumed she might have pseudopacemaker syndrome. To correct this, the PVARP was shortened from 250 to 210 ms, and the pacemaker was switched to DDD mode. Metoprolol 25 mg daily was initiated for short episodes of atrial tachycardia.

At 4 months postpacemaker implantation, treadmill stress echocardiography was performed to evaluate chest discomfort and chronotropic competence. The patient exercised for 9 min and 41 s on the Bruce protocol, reaching 11.6 metabolic equivalents (METs), with a heart rate of 146 bpm (83% of predicted max) with a peak blood pressure of 145/62 mmHg. She experienced mild, nonlimiting chest discomfort during exercise but no significant dyspnea. Baseline ECG showed sinus rhythm with RBBB and no ischemic changes; rare isolated premature ventricular contractions were noted. The test was terminated due to a precipitous drop in heart rate at peak exertion. Echocardiography revealed a normal resting EF, appropriate augmentation with exercise, and normalization during recovery. The findings indicated a normal stress echocardiogram without ischemia but revealed an abrupt heart‐rate drop consistent with pacemaker Wenckebach secondary to upper tracking‐rate limiting higher heart rate beyond the set rate. The patient underwent reprogramming to address this “rate ceiling.” The upper tracking rate was increased from 145 to 165 bpm, and the upper sensor rate from 135 to 155 bpm. These changes were intended to prevent rate‐limiting pseudoblock at higher sinus rates and improve exercise tolerance. The patient subsequently experienced improvement in her symptoms and reported renewed stamina and energy. Metoprolol was discontinued due to lightheadedness and low blood pressure.

At 6 months postprocedure, she still described mild exertional fatigue without pain. Interrogation showed atrial pacing 57%, ventricular pacing 100%, and stable lead parameters. To enhance chronotropy, the device was reprogrammed to DDDR mode with an accelerated rate‐response profile, maintaining the upper limits of 165 bpm (tracking) and 155 bpm (sensor). Thyroid, hematologic, and metabolic studies were normal. A repeat treadmill test around 7 months postprocedure confirmed physiologic rate response: 9 min 19 s of Bruce protocol (≈10 METs), peak heart rate 151 bpm (85% of predicted), blood pressure 150/77 mmHg, mild early chest discomfort improving with exercise, and no ischemic ECG changes, consistent with normalization of chronotropic function.

**Figure 3:**
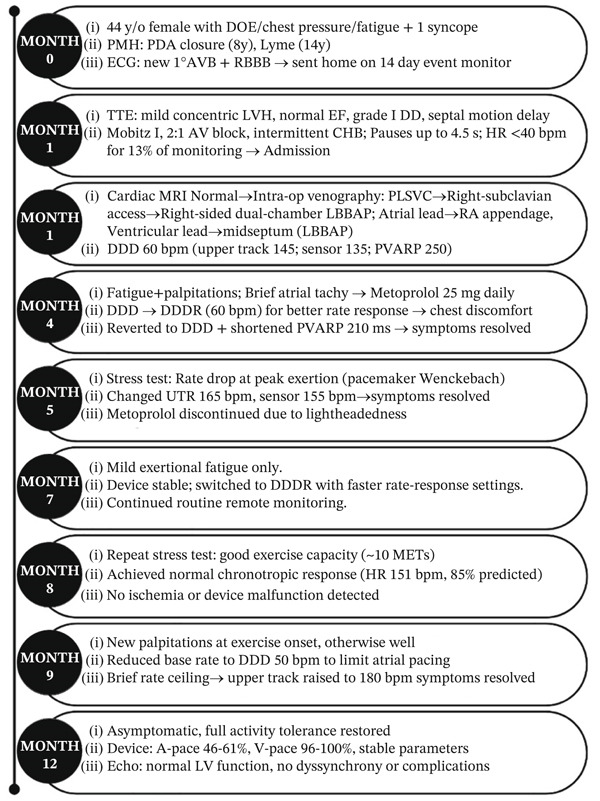
Timeline of clinical presentation, diagnostic evaluation, and management.

At 9 months, she reported palpitations at the start of exercise but was otherwise healthy. Finally, the device was programmed to DDD at 50 bpm to reduce unnecessary atrial pacing, and the tracking rate was increased from 165 to 180 bpm to meet her physiological needs. By 1 year, she resumed full activities without syncope, chest discomfort, or device issues (Figure 3).

## 3. Discussion

In this case, symptomatic high‐grade atrioventricular block and intraoperative identification of PLSVC prompted a change in procedural strategy from a traditional left‐sided to a right‐sided approach. Although hemodynamically silent, PLSVC can make conventional transvenous lead delivery difficult because the coronary sinus route brings acute angulations and poorer mechanical support for sheaths and leads. LBBAP is a physiologic pacing technique that requires precise septal penetration to capture the left bundle or its fascicles. This requires perpendicular orientation of the pacing sheath against the interventricular septum, an objective that is more easily achieved through standard right‐sided venous access. In patients with PLSVC, conventional left‐sided approaches may not allow optimal sheath positioning or lead stability, potentially precluding successful deployment of conduction system pacing. Because the communicating channel to the right SVC arose at an acute angle, a left‐sided approach was considered less favorable for conduction system pacing. A right‐sided dual‐chamber pacemaker with LBBAP was therefore implanted successfully using a standard fixed‐curve sheath designed for a left‐sided approach and a lumenless Medtronic 3830 lead. To our knowledge, reports of right‐sided LBBAP in PLSVC remain limited [7, 8], and co‐occurrence of PLSVC with a communicating channel to the right SVC in this context is rarely described.

The recently reported pooled prevalence of PLSVC is 0.61%, with bilateral superior vena cava representing the most frequently quantified major configuration and isolated PLSVC occurring substantially less often [3]. These anatomical distinctions are procedurally important because the presence of a right superior vena cava or bridging brachiocephalic vein may preserve alternative routes for transvenous lead implantation, whereas isolated PLSVC may require implantation through the coronary sinus or another individualized strategy.

Bulava et al. [7] reported successful LBBAP through a PLSVC using a stylet‐driven lead and substantial manipulation through the coronary sinus. McGee et al. [9] described left bundle branch pacing through the left brachiocephalic vein in a patient with persistent left‐sided SVC. Deshmukh et al. [1] described successful His‐bundle pacing in a patient with isolated PLSVC using a left‐sided approach via the coronary sinus with a steerable sheath and mapping catheter.

Prolič Kalinšek and Žižek [10] demonstrated right‐sided LBBAP in a patient with PLSVC with manual modification of the C315‐His sheath to obtain appropriate septal contact. Although their experience showed feasibility, they also highlighted the need for sheath modification. Ponnusamy et al. [11] also showed that right‐sided conduction‐system pacing can be achieved with standard LBBAP tools in the presence of complex venous anatomy. Also, Menemencioglu and Canpolat [8] demonstrated successful right‐sided CRT delivery via LBBAP in a PLSVC patient, though their case involved heart failure and necessitated cardiac resynchronization. Like these reports, our case also supports a right‐sided approach for bradycardia pacing in a structurally normal heart using standard equipment.

**Figure 4:**
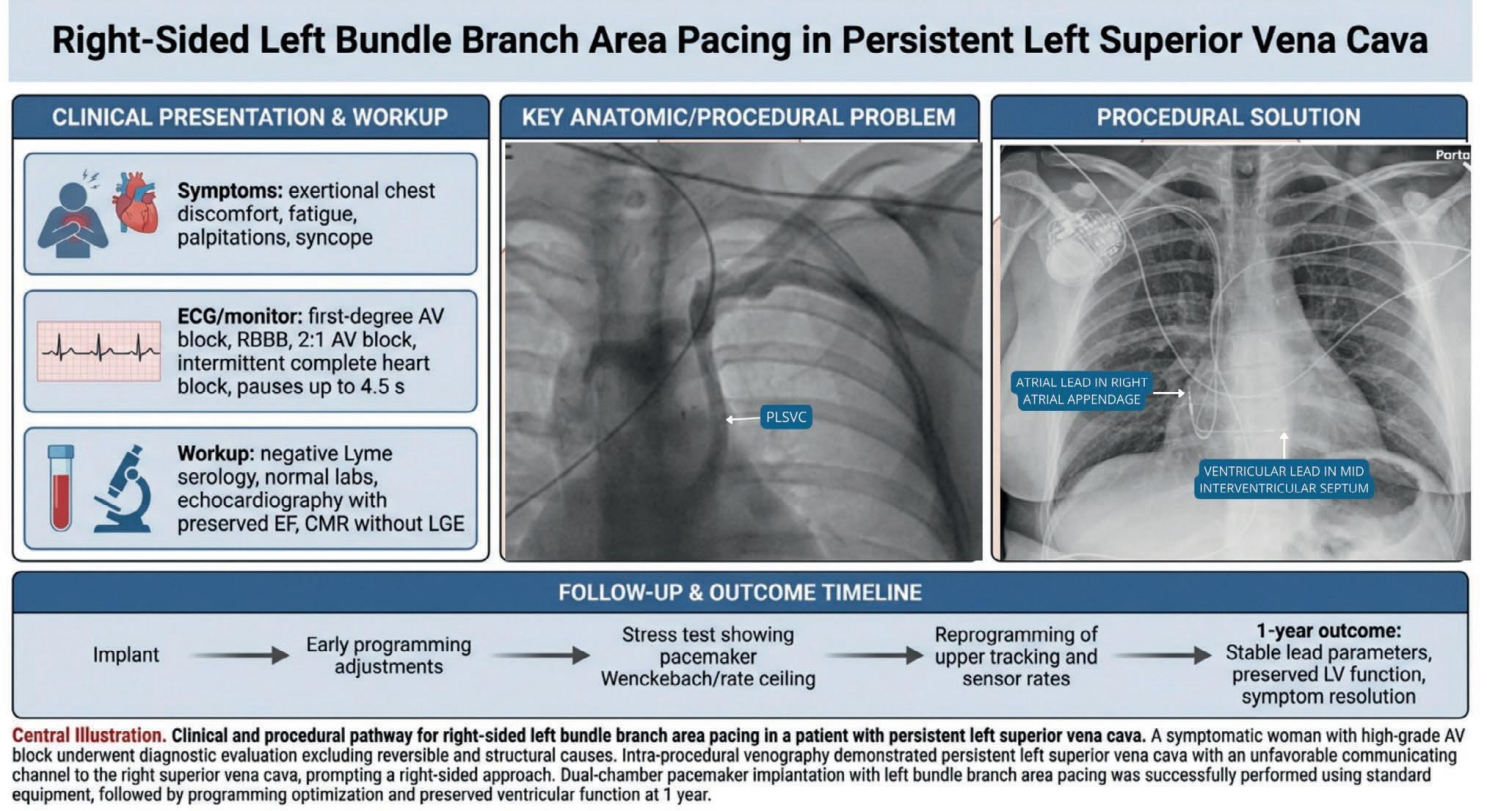
Central illustration.

The clinical relevance of this approach is important in younger patients expected to require lifelong ventricular pacing. In our patient, preserved left ventricular function and high anticipated pacing burden made physiologic pacing preferable to right ventricular apical pacing, which may increase the risk of pacing‐induced cardiomyopathy over time. Right‐sided LBBAP preserved ventricular synchrony while avoiding the technical difficulty of left‐sided lead delivery. At 1‐year follow‐up, the patient had stable lead parameters, preserved ventricular function, and complete symptom resolution with optimized personalized pacing parameters compared with the default pacemaker setting (Figure 4).

## 4. Conclusions

In young patients with bradycardia who are expected to require substantial ventricular pacing, physiologic pacing should be prioritized to preserve ventricular synchrony and reduce the risk of ventricular dysfunction. This case demonstrates that a PLSVC does not preclude conduction‐system pacing and that right‐sided LBBAP can be safely and effectively achieved with standard sheaths and leads in anatomically suitable patients, thereby avoiding more complex, customized approaches. In selected patients, right‐sided LBBAP offers a safe, technically feasible, and effective alternative when conventional left‐sided approaches are anatomically limited. Importantly, once adequate septal lead deployment is achieved, long‐term electrical performance and clinical outcomes appear comparable to traditional pacing systems. Lastly, appropriate programming of the device is crucial, considering age, gender, and physiologic needs based on activity level to avoid symptoms related to pacemaker behaviors and recovery.

**Nomenclature**
AV: atrioventricular
CMR: cardiac magnetic resonance
ECG: electrocardiogram
LBBAP: left bundle branch area pacing
LGE: late gadolinium enhancement
LVEF: left ventricular ejection fraction
PLSVC: persistent left superior vena cava
PVARP: postventricular atrial refractory period
RBBB: right bundle branch block
SVC: superior vena cava

## Funding

No funding was received for this manuscript.

## Disclosure

With regard to provenance and peer review, the work was not commissioned and was externally peer‐reviewed.

## Ethics Statement

Patient consent for publication: Written informed consent has been obtained from the patient for publication of this case report and any accompanying images. Institutional ethical review is not required, as this is a case report; therefore, approval from the institutional review committee was not obtained.

## Conflicts of Interest

The authors declare no conflicts of interest.

## Data Availability

The data that support the findings of this study are available from the corresponding author upon reasonable request.
